# *Alternaria alternata* (Fr) Keissl Crude Extract Inhibits HIV Subtypes and Integrase Drug-Resistant Strains at Different Stages of HIV Replication

**DOI:** 10.3390/ph18020189

**Published:** 2025-01-30

**Authors:** Darian Naidu, Ernest Oduro-Kwateng, Mahmoud E. S. Soliman, Sizwe I. Ndlovu, Nompumelelo P. Mkhwanazi

**Affiliations:** 1 HIV Pathogenesis Programme, School of Laboratory Medicine and Medical Science, College of Health Science, University of KwaZulu-Natal, Durban 4041, South Africa; 218052764@stu.ukzn.ac.za; 2 Molecular Bio-Computation and Drug Design Research Group, School of Health Sciences, University of KwaZulu Natal, Durban 4041, South Africasoliman@ukzn.ac.za (M.E.S.S.); 3 Unit for Environmental Sciences and Management, North-West University, Potchefstroom 2520, South Africa; sizwe.ndlovu@nwu.ac.za

**Keywords:** *A. alternata*, anti-HIV activity, molecular docking, integrase inhibitors, HIV-subtypes

## Abstract

**Background/Objectives**: The development of HIV drug resistance to current antiretrovirals, and the antiretrovirals’ inability to cure HIV, provides the need of developing novel drugs that inhibit HIV-1 subtypes and drug-resistance strains. Fungal endophytes, including *Alternaria alternata*, stand out for their potentially antiviral secondary metabolites. Hence, this study investigates the anti-HIV activities and mechanism of action of the *A. alternata* crude extract against different HIV-1 subtypes and integrase-resistant mutant strains. **Methods**: Cytotoxicity of the *A. alternata* crude extract on TZM-bl cells using the 3-(4,5-dimethylthiazol-2-yl)-2,5-diphenyl tetrazolium bromide (MTT) assay was performed. The crude extract antiviral activity against subtypes A, B, C, and D and integrase drug-resistant strain T66K and S230R was determined using a luciferase-based antiviral assay. Luciferase and p24 ELISA-based time-of-addition assays were used to determine the mechanism of action of the crude extract. Docking scores and protein ligand interactions of integrase T66K and S230R strains against the identified bioactive compounds were determined. **Results**: The crude extract CC_50_ was 300 μg/mL and not cytotoxic to the TZM-bl cell lines. In HIV-1 subtypes A, B, C, and D, the crude extract exhibited 100% inhibition and therapeutic potential. The *A. alternata* crude extract had strong anti-HIV-1 activity against integrase strand transfer drug-resistant strains T66K and S230R, with a 0.7265- and 0. 8751-fold increase in susceptibility. The crude extract had antiviral activity during attachment, reverse transcription, integration, and proteolysis. In silico calculations showed compounds 2,3-2H-Benzofuran-2-one, 3,3,4,6-tetramethyl-, 3-Methyl-1,4-diazabicyclo[4.3.0]nonan-2,5-dione, N-acetyl, Coumarin, 3,4-dihydro-4,5,7-trimethyl-, Cyclopropanecarboxamide, N-cycloheptyl, Pyrrolo[1,2-a]pyrazine-1,4-dione, and hexahydro-3-(2-methylpropyl)- crude extract bioactive compounds had strong docking scores and diverse binding mechanisms with integrase. **Conclusions**: The *A. alternata* crude extract demonstrates strong antiviral activity against different HIV-1 subtypes and integrase drug-resistance strains. The extract inhibited various stages of the HIV-1 life cycle. The bioactive compounds 2,3-2H-Benzofuran-2-one, 3,3,4,6-tetramethyl-, 3-Methyl-1,4-diazabicyclo[4.3.0]nonan-2,5-dione, N-acetyl, Coumarin, 3,4-dihydro-4,5,7-trimethyl-, Cyclopropanecarboxamide, N-cycloheptyl, Pyrrolo[1,2-a]pyrazine-1,4-dione, and hexahydro-3-(2-methylpropyl)- may be responsible for the antiviral activity of *A. alternata*.

## 1. Introduction

According to UNAIDS [1], 39.9 million people are living with HIV/AIDS (PLWH) worldwide. AIDS-related mortality and co-morbidities have been successfully decreased with antiretrovirals [1]. Antiretroviral drugs successfully reduced the morbidity and mortality rate of PLWH by turning HIV/AIDS into a chronic and manageable illness [2,3]. Notably, HIV-1 is diverse and highly mutational, which results in the fast development of drug resistance. It comprises nine subtypes, making antiretroviral drugs respond differently [4,5]. However, antiretrovirals are still disadvantaged by challenges such as renal dysfunction and weight gain, HIV-1 subtype diversity, and the emergence of drug resistance rendering them no longer curative [6,7,8,9,10]. Thus, there is a research gap regarding HIV therapeutics that do not share the limitations of modern antiretrovirals.

Natural products (NPs) are being explored as a novel strategy to address antiretroviral challenges [11,12]. The scientific community has also attempted to address this research gap by bioprospecting NPs from medicinal plants such as *Hypoxis hemerocallidea*, which are used in traditional medicine to treat HIV [13,14]. An early phase 1 clinical trial of an *H. hemerocallidea* methanolic extract increased the survival of PLWH by years and decreased serum p24 [15]. Medicinal plants are under threat of over-harvesting, which has the potential to cause irreversible environmental damage [13,16]. Due to these challenges, the attention has shifted to fungal endophytes of medicinal plants such as *A. alternata*, which have the potential to share medicinal properties of the plant host and be a more sustainable strategy to produce and study anti-HIV NPs [17,18,19].

Previous studies have shown that SMs from endophytic fungi may possess anti-HIV activity [19,20,21]. A study conducted by Wellensiek et al. [21] showed that a fractionated crude extract of *Alternaria tenuissima* was a potent inhibitor of HIV reverse transcriptase and HIV-1 viral replication. However, the active compounds were not identified [20]. Subsequently, altertoxins isolated from *Alternaria tenuissima* were found to be potent inhibitors of HIV with 0.09–1.42 μM of IC50 [22]. However, these alter toxins were found to have a low therapeutic index, but their epoxyperylene scaffold may be a good candidate for further drug development [22,23]. Furthermore, coumarin-type compounds isolated from *Alternaria species* inhibited reverse transcription and gp120 binding by 82.81% and 86.38%, respectively [24]. Recently, analogues of calanolide A, a coumarin-type compound, have been used as a scaffold to synthesise anti-HIV drugs [25].

Natural products have been explored alongside existing approaches as a novel strategy to inhibit the replication of HIV [11,12]. Recently, the *A. alternata* crude extracts isolated from the *Hypoxis* species were found to have an IC_50_ of 0.017–1.170 μg/mL against HIV-1 subtype B (pNL4.3) [19]. Nzimande et al. [19] established that the secondary metabolites of A. *alternata* have strong anti-HIV activity. However, they did not determine the mechanism of action, the activity of non-subtype B viruses, or drug-resistant strains. Recently, it has also been reported that fractioned extracts of *A. alternata* were potent inhibitors of HIV-1 subtype B and had viral attachment, reverse transcription, integration, and proteolysis inhibitory activity [26]. However, Kubheka et al. [26] did not establish the anti-HIV effects on different HIV-1 subtypes and integrase drug-resistant strains. Therefore, this study investigated the antiviral effect of the *A. alternata* crude extract on different HIV-1 subtypes, integrase drug-resistant strains by luciferase-based antiviral assays, and the mechanism of action by time-of-addition assay. Molecular docking was used to determine the binding affinity of the bioactive compounds to the HIV-1 integrase enzyme and provide insights into their molecular mechanics of inhibition. This study focuses on integrase due to incorporating INSTIs in South African regimens and the rise of INSTI drug resistance in PLWHs. The *A. alternata* crude extract has been tested on wild-type HIV, and we want to investigate the activity on drug-resistant strains [19,26]. Therefore, this study investigated the anti-HIV activities and mechanism of action of secondary metabolites from *A. alternata* on different HIV-1 subtypes and integrase-resistant mutant strains.

This study adds to the progress made by Nzimande et al. [19] and Kubheka et al. [26] studying *A. alternata*. Previously, we only investigated the antiviral activity of the *A. alternata* crude extracts against the HIV subtype B virus. Here, we investigated the antiviral activities of the *A. alternata* crude extracts against subtype A, B, C, and D viruses and drug-resistant strains T66K and S230R. Kubheka et al. [26] only investigated the mechanism of action on HIV subtype B. However, this study also investigated the mechanism of action on subtypes A, B, C, and D. Nzimande et al. [19] conducted A GC-MS analysis of the *A. alternata* crude extract. The GC-MS data (Table S1) from Nzimande et al. [19] were used for molecular docking and protein–ligand interaction analysis. In summary, this study did make progress by highlighting the efficacy of the *A. alternata* crude extract against drug-resistant strains. It investigated potential mechanisms of HIV integrase inhibition using molecular docking and protein–ligand interaction analysis. The findings of this study hope to address challenges such as drug toxicity, INSTI drug resistance, and HIV subtype diversity using natural products such as endophytic *A. alternata*. In addition, this study encourages further research into natural products to address HIV challenges and potentially inspire future drug design. *A. alternata*’s secondary metabolites may possess multi-target HIV-1 antiretroviral therapy.

## 2. Results

### 2.1. Cytotoxicity and Cell Viability of A. alternata CE Using MTT

**Figure 1:**
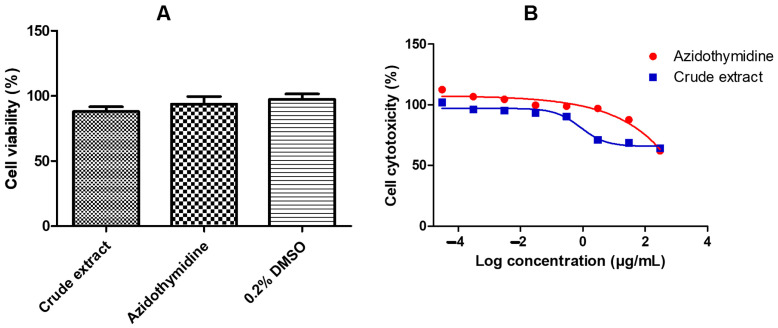
Cell viability (**A**) and cell cytotoxicity (**B**) of the *A. alternata* crude extract were determined by MTT assay. The cell viability (**A**) *y*-axis represents the percentage cell viability of the TZM-bl cells and the *x*-axis represents the treatments crude extract, azidothymidine, and 0.2% DMSO. The TZM-bl cells treated dose-response curves (**B**) showing the percentage cell cytotoxicity were used to determine the CC_50_ of the *A. alternata* crude extract blue line (CC_50_ = 300 ± 1.83 µg/mL) and azidothymidine red line (CC_50_ = 385 ± 6.29 µg/mL). The *y*-axis of the dose-response curves shows the percentage of cell cytotoxicity and the *x*-axis shows the log concentration of the *A. alternata* crude and azidothymidine. The MTT assay was completed in triplicate.

Figure 1A illustrates that the viability of the *A. alternata* crude extract is above 80% using MTT (3-(4,5-dimethylthiazol-2-yl)-2,5-diphenyltetrazolium bromide) assay t on the TZM-bl cell line. Figure 1B shows that the CC_50_ of the crude extract was 300 ± 1.83 ug/mL. The cell viability (Figure 1A) and cytotoxicity (Figure 1B) of the drug control azidothymidine and 0.2% DMSO were also greater than 80%.

### 2.2. Antiviral Activity of A. alternata Crude Extract Against Different HIV-1 Subtypes

**Figure 2:**
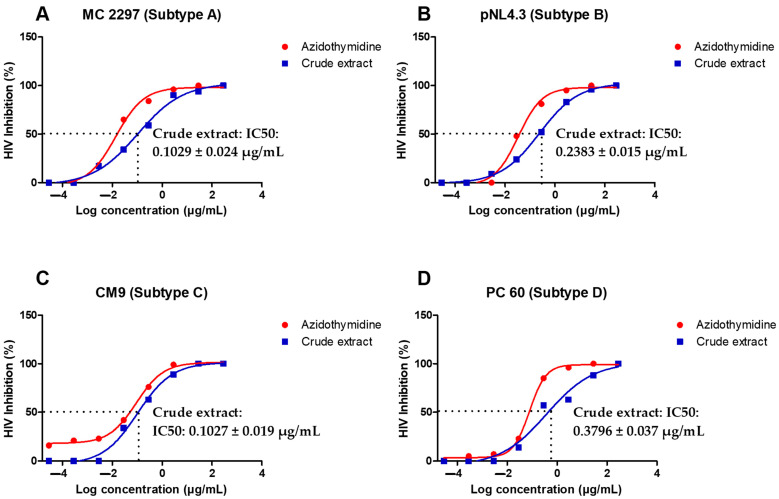
Dose-dependent curves showing antiviral activity of *A. alternata* crude extract against MC 2297 (**A**), pNL4.3 (**B**) CM9 (**C**), and PC 60 (**D**) HIV-1 viruses belonging to respective subtypes A, B, C, and D. The *y*-axis of (**A**–**D**) shows the inhibition of HIV as a percentage. The *x*-axis (**A**–**D**) shows the log concentration of the crude extract or azidothymidine. The red line shows the positive control azidothymidine and the blue line shows the *A. alternata* crude extract. The luciferase-based antiviral assay was completed in triplicate.

The *A. alternata* crude extract antiviral activity was evaluated using a luciferase-based antiviral assay against TZM-bl cells infected with PC 148 (subtype A), MC 2297 (subtype A), pNL4.3 (subtype B), YU2 (subtype B), CM7 (subtype C), CM9 (subtype C), PC 60 (subtype D), and PC 178 (subtype D). One dose-dependent curve of one virus from each subtype was presented graphically (Figure 2A–D). The reverse transcriptase inhibitor azidothymidine was used as a positive control. An IC50 less than 10 µg/mL indicates the treatment was a highly active inhibitor of HIV-1. The IC50s of PC 148, MC 2297, pNL4.3, YU2, CM7, CM9, PC 60, and PC 178 treated with the *A. alternata* crude extract in (Figure 2A–D) were less than 10 µg/mL, meeting the requirements to be considered highly active against the subtype A, B, C, and D viruses.

### 2.3. Analysis of Half-Maximal Inhibitory Concentrations and Selective Indexes of A. alternata Crude Extract Against HIV-1 Subtypes A, B, C, and D Viruses

The IC50 and SI data for the *A. alternata* crude extract and azidothymidine drug control against PC 148, MC 2297, pNL4.3, YU2, CM7, CM9, PC 60, and PC 178 HIV-1 viruses were summarised in (Table 1). Selective indexes were calculated as the ratio CC_50_/IC_50_. An SI greater than 10 indicates that the treatment or sample has therapeutic potential [27]. 

### 2.4. Relative Fold Change in IC50s of A. alternata Crude Extract Against Different HIV-1 Subtypes

**Figure 3:**
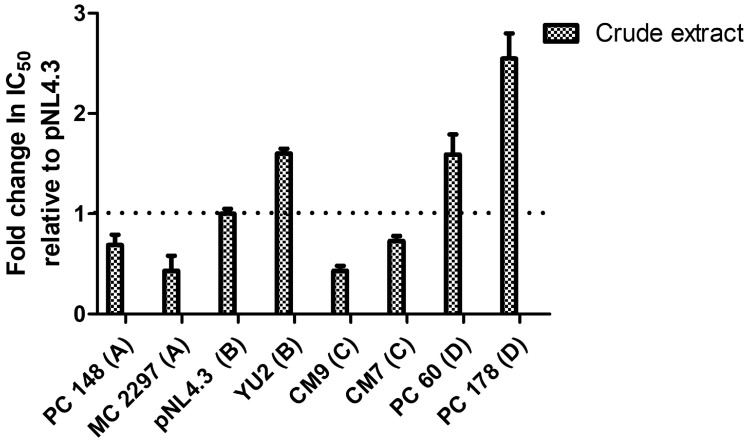
The fold change in IC_50_ of viruses PC 148, MC 2297, pNL4.3, YU2, CM7, CM9, PC 60, and PC 178 treated with the *A. alternata* crude extract relative to pNL4.3. The *y*-axis represents the fold change in IC50 relative to pNL4.3. The *x*-axis represents the virus treated with the *A. alternata* crude extract. The blue bars indicate that *A. alternata* crude extract was the treatment used. The luciferase-based antiviral assay was completed in triplicate.

The IC50s of the PC 148, MC 2297, pNL4.3, YU2, CM7, CM9, PC 60, and PC 178 HIV-1 subtype A, B, C, and D viruses were expressed as a fold change in IC50 relative to the IC50 of pNL4.3 in (Figure 3) since pNL4.3 is known to be infectious. The fold change in IC50, relative to control pNL4.3, was calculated as a ratio of virus subtype IC50/pNL4.3 IC50. A fold change in IC50 less than 1 indicates an increase in IC50, indicating greater susceptibility.

### 2.5. Confirmation of Mutations Generated by Site-Directed Mutagenesis

The full-length integrase gene sequences of the site-directed mutants were assembled in (Figure S1). The assembled sequences were aligned with the wild-type integrase gene to confirm the presence of the integrase drug-resistance mutations. In (Figure S2A–D), the mutations T66K, E92Q, S230R, and R263K were confirmed.

### 2.6. Infectivity of HIV-1 Subtypes and Integrase Drug-Resistant Strains T66K, E92Q, S230R, and R263K

**Figure 4:**
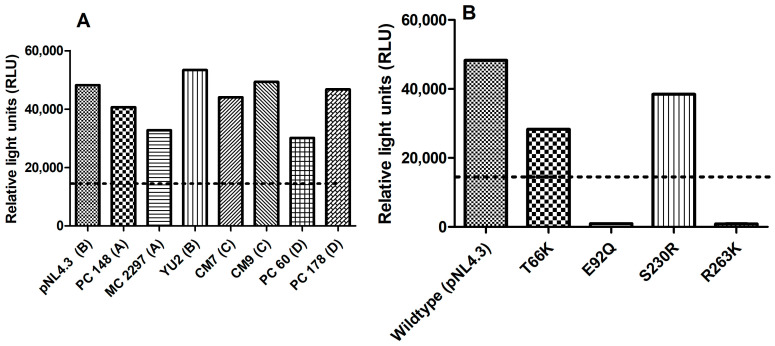
The infectivity of different subtype HIV-1 viruses PC 148, MC 2297, YU2, CM7, CM9, PC 60, and PC 178 (**A**) T66K, E92Q, S230R, and R263K (**B**) integrase drug-resistant mutants relative to control wild-type virus pNL4.3. The *y*-axis represents the infectivity of (**A**,**B**) viruses in relative light units (RLUs). The *x*-axis of (**A**,**B**) represents the virus being tested.

The infectivity of the different subtype HIV-1 viruses PC 148, MC 2297, YU2, CM7, CM9, PC 60, and PC 178, as well as T66K, E92Q, S230R, and R263K integrase drug-resistant mutants in TZM-bl cells in Figure 4A,B, were expressed as relative light units (RLUs) against the wild-type virus pNL4.3. The integrase drug-resistant HIV strains were considered infectious if they were able to produce at least 15,000 RLUs. The different subtype HIV viruses (Figure 4A), as well as integrase drug-resistant HIV strains T66K and S230R (Figure 4B), were considered infectious since both viruses had produced more than 15,000 RLUs.

### 2.7. Antiviral Activity of A. alternata Crude Extract Against Integrase Drug-Resistant Mutants

**Figure 5:**
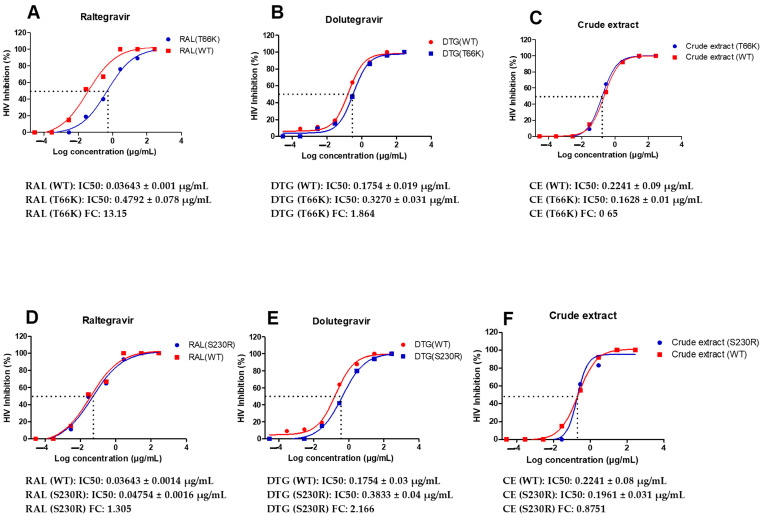
Dose-response curves of *A. alternata* crude extract integrase drug-resistant strains T66K and S230R. The *y*-axis of (**A**–**F**) shows the inhibition of HIV as a percentage. The *x*-axis (**A**–**F**) shows the log concentration of the *A. alternata* crude extract, raltegravir, or dolutegravir. The red lines (**A**,**D**), (**B**,**E**), and (**C**,**F**) show raltergravir (WT), dolutergravir (WT), and *A. alternata* crude extract (WT), respectively. The blue lines (**A**–**C**) and (**D**–**F**) show raltergravir (T66K), dolutergravir (T66K), *A. alternata* crude extract (T66K), raltergravir (S230R), dolutergravir (S230R), and *A. alternata* crude extract (S230R), respectively. The luciferase-based antiviral assay was completed in triplicate.

The antiviral activity of the *A. alternata* crude extract and integrase inhibitor drug controls raltegravir (RAL) and dolutegravir (DTG) was evaluated against the integrase inhibitor drug-resistance strains T66K and S230R using a luciferase-based assay (Figure 5). At 300 µg/mL, raltegravir, dolutegravir, and the *A. alternata* crude extract elicited 100% inhibition of HIV replication of T66K and S230R drug-resistant strains. The fold change (FC) was then calculated as the ratio of drug-resistant strain IC_50_/wild-type IC_50_. Therefore, if there is no change in the drug-resistant strain IC_50_, then the fold change will equal 1. If drug-resistant strain IC_50_ is greater than the wild type then the fold change will be greater than 1, which indicates resistance. If the drug-resistant strain IC_50_ is less than the wild type, then the fold change will be less than 1, which indicates susceptibility.

### 2.8. Luciferase-Based Time of Addition

**Figure 6:**
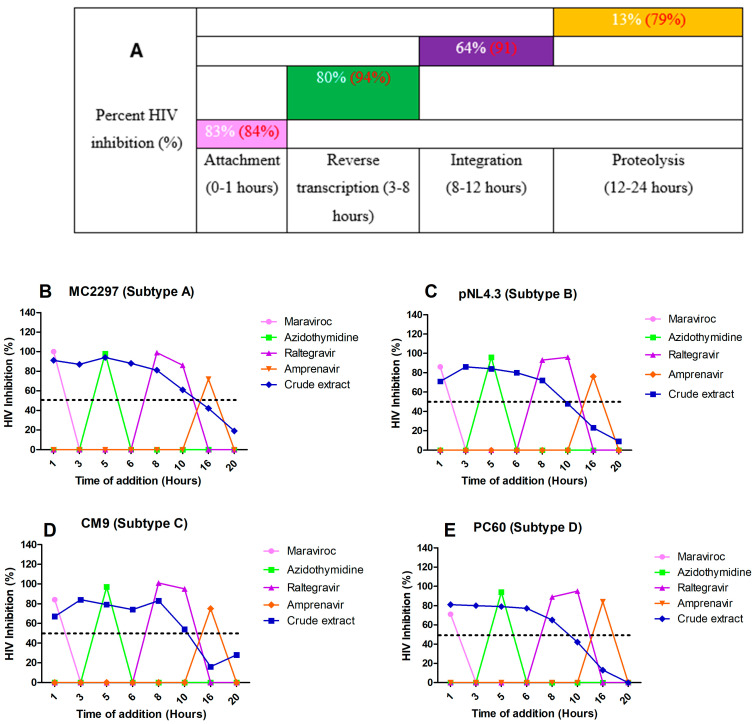
The overall inhibition of the subtype A, B, C, and D viruses treated with *A. alternata* crude extract during different stages in the HIV life cycle (**A**). For (**A**), the *y*-axis represents the inhibition as a percentage and the *x*-axis represents the stage of the HIV life cycle. The pink, green, purple, and orange squares represent attachment, reverse transcription, integration, and proteolysis, respectively. The red text represents the percentage inhibition of the drug controls maraviroc, azidothymidine, raltegravir, and amprenavir, respectively. The white text represents the percentage inhibition of *A. alternata* crude extract. Luciferase-based time of addition of viruses MC2297 (**B**), pNL4.3 (**C**), CM9 (**D**), and PC60 (**E**). The *y*-axis of (**B**–**E**) shows the inhibition of HIV as a percentage. The *x*-axis of (**B**–**E**) shows the time of addition in hours. For (**B**–**E**), the pink, green, purple, orange, and blue lines of (**B**–**E**) represent maraviroc, azidothymidine, raltegravir, amprenavir, and *A. alternata* crude extract, respectively. The *A. alternata* crude extract was added at 1, 3, 5, 6, 8, 10, 16, and 20 h post-infection. The luciferase-based time-of-addition assay was done in duplicate.

The principle of time of addition is that key stages in the life cycle of HIV occur during attachment (0–1 h), reverse transcription (3–8 h), integration (6–12 h), and proteolysis (16–24 h). A luciferase-based time-of-addition assay was used to determine the mechanism of action of the *A. alternata* crude extract. The attachment, reverse transcription, integrase, and protease inhibitors maraviroc, azidothymidine, raltegravir, and amprenavir were used as drug controls for each key stage of the HIV life cycle. The *A. alternata* crude extract was considered to have activity if the percentage of HIV inhibition was 50% or more at a time interval. The luciferase-based time-of-addition assay was performed on viruses PC 148, MC 2297, YU2, CM7, CM9, PC 60, and PC 178 (Figure 6), as well as integrase drug-resistant strains T66K and S230R (Figure 6). However, one virus from each subtype was shown.

The HIV-1 subtype A viruses (Figure 6B) treated with the *A. alternata* crude extract had inhibitory activity during attachment (88%), reverse transcription (80%), integration (63%), and proteolysis (16%). The HIV-1 subtype B (Figure 6C) viruses treated with the *A. alternata* crude extract had inhibitory activity during attachment (79%), reverse transcription (78%), integration (64%), and proteolysis (11%). The HIV-1 subtype C (Figure 6D) viruses treated with the *A. alternata* crude extract had inhibitory activity during attachment (83%), reverse transcription (84%), integration (69%), and proteolysis (19%). The HIV-1 subtype D (Figure 6E) viruses treated with the *A. alternata* crude extract had inhibitory activity during attachment (86%), reverse transcription (79%), integration (65%), and proteolysis (5%).

**Figure 7:**
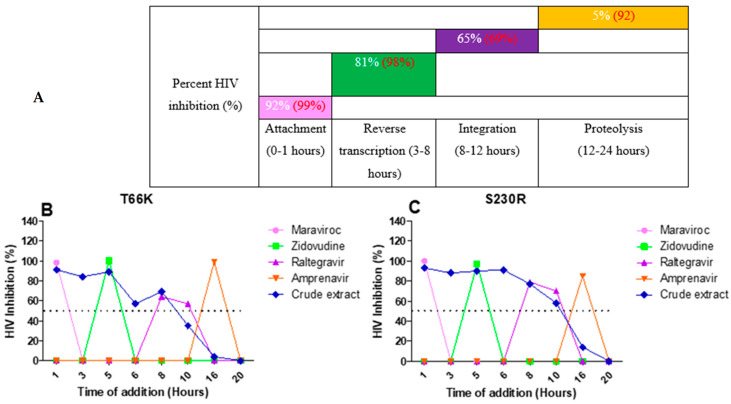
The overall inhibition of the integrase drug-resistant viruses treated with *A. alternata* crude extract during different stages in the HIV life cycle (**A**). For (**A**), the *y*-axis represents the inhibition as a percentage and the *x*-axis represents the stage of the HIV life cycle. The pink, green, purple, and orange squares represent attachment, reverse transcription, integration, and proteolysis, respectively. The red text represents the percentage inhibition of the drug controls maraviroc, azidothymidine, raltegravir, and amprenavir, respectively. The white text represents the percentage inhibition of *A. alternata* crude extract. Luciferase-based time of addition of integrase drug-resistant viruses T66K (**B**) and S230R (**C**). The *y*-axis of (**B**,**C**) shows the inhibition of HIV as a percentage. The *x*-axis of (**B**,**C**) shows the time of drug addition in hours. For (**B**,**C**), the pink, green, purple, orange, and blue lines of (**B**,**C**) represent maraviroc, azidothymidine, raltegravir, amprenavir, and *A. alternata* crude extract, respectively. The *A. alternata* crude extract was added at 1, 3, 5, 6, 8, 10, 16, and 20 h post-infection. The luciferase-based time-of-addition assay was done in duplicate.

The integrase drug-resistant virus T66K (Figure 7B) treated with the *A. alternata* crude extract had inhibitory activity during attachment (91%), reverse transcription (87%), integration (54%), and proteolysis (2%). The integrase drug-resistant virus S230R (Figure 7C) treated with the *A. alternata* crude extract had inhibitory activity during attachment (93%), reverse transcription (89%), integration (75%), and proteolysis (7%).

### 2.9. HIV-1 p24-Based Time-of-Addition Assay

HIV-1 p24-based time-of-addition assay was used to determine the mechanism of action of the *A. alternata* crude extract. The viruses CM9 and MC2297 were selected for the p24-based time-of-addition assay since both displayed IC50s with the highest anti-HIV activity. The integrase drug-resistant viruses T66K and S230R were also selected as integrase drug-resistant viruses to undergo the p24-based time-of-addition assay. For viruses CM9, MC2297, and S230R, an HIV-1 p24 titre equal to 40 pg/mL or less was used to indicate the antiviral activity of the *A. alternata* crude extract. For the virus T66K, due to a weaker HIV-1 p24 titre, a titre equal to 20 pg/mL or less was used to indicate the antiviral activity of the *A. alternata* crude extract.

**Figure 8:**
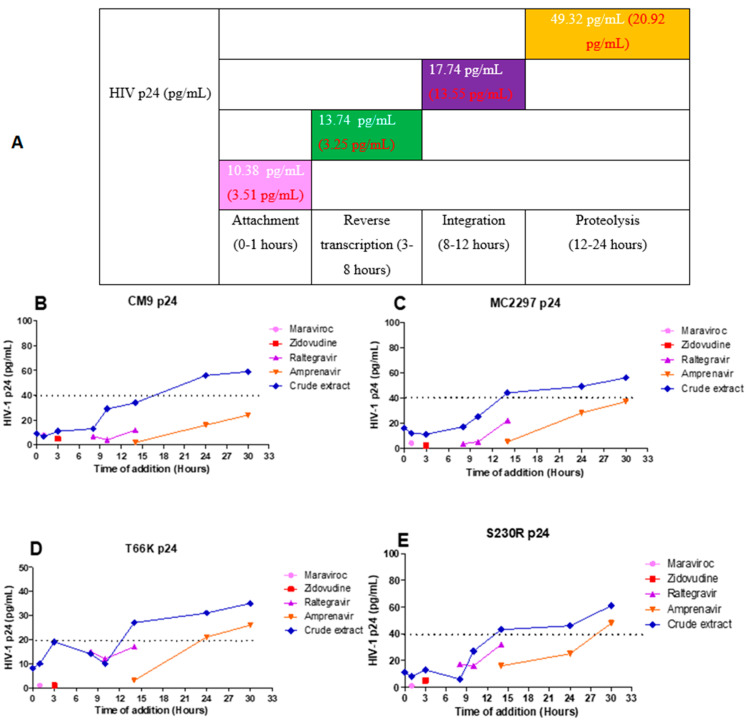
The overall HIV p24 titre (pg/mL) of the CM9, M2297, T66K, and S230R viruses treated with *A. alternata* crude extract during different stages in the HIV life cycle (**A**). The pink, green, purple, orange, and blue lines of (**B**–**E**) represent maraviroc, azidothymidine, raltegravir, amprenavir, and *A. alternata* crude extract, respectively. For (**A**), the *y*-axis represents the inhibition as a percentage and the *x*-axis represents the stage of the HIV life cycle. The pink, green, purple, and orange squares represent attachment, reverse transcription, integration, and proteolysis, respectively. The red text represents the percentage inhibition of the drug controls maraviroc, azidothymidine, raltegravir, and amprenavir, respectively. The white text represents the percentage inhibition of the *A. alternata* crude extract. p24-based time of addition of viruses CM9 (**B**), M2297 (**C**), T66K (**D**), and S230R (**E**). The *y*-axis of (**B**–**E**) shows the concentration of HIV p24 in pg/mL. The *x*-axis of (**B**–**E**) shows the time of addition in hours. The *A. alternata* crude extract was added at 0, 1, 3, 8, 10, 14, 24, and 30 h post-infection. The pink, green, purple, and orange squares represent attachment, reverse transcription, integration, and proteolysis, respectively. The HIV-1 p24 time-based ELISA was completed once due to budget constraints. Therefore, a luciferase-based time-of-addition assay was done with the HIV-1 p24 time-based ELISA.

The HIV-1 subtype A virus MC2297 (Figure 8B) treated with the *A. alternata* crude extract had inhibitory activity during attachment (14.53 pg/mL), reverse transcription (14.24 pg/mL), integration (21.19 pg/mL), and proteolysis (49.85 pg/mL). The HIV-1 subtype C virus CM9 (Figure 8C) treated with the *A. alternata* crude extract had inhibitory activity during attachment (8.04 pg/mL), reverse transcription (12.20 pg/mL), integration (20.94 pg/mL), and proteolysis (49.67 pg/mL). The INSTI drug-resistant virus T66K (Figure 8D) treated with the *A. alternata* crude extract had inhibitory activity during attachment (9.28 pg/mL), reverse transcription (16.69 pg/mL), integration (12.34 pg/mL), and proteolysis (31.25 pg/mL). The integrase drug-resistant virus S230R (Figure 8E) treated with the *A. alternata* crude extract had inhibitory activity during attachment (9.66 pg/mL), reverse transcription (9.68 pg/mL), integration (16.49 pg/mL), and proteolysis (50.28 pg/mL).

### 2.10. Docking Calculations of the Bioactive Compounds of A. alternata Against HIV-1 Integrase Strains (Wild Type, T66K, and S230R)

The physiological function of the active site of HIV-1 integrase is vital for the integration of viral DNA into the host genome, enabling the replication and persistence of the virus within the host [28]. Therefore, we performed docking calculations at the active site of the protein targets with the bioactive compounds of *A. alternata*. The docking studies provide a comparative analysis of the binding affinities of the secondary metabolites against HIV-1 integrase wild-type and resistant strains (T66K and S230R), as well as their performance relative to the reference integrase inhibitors, raltegravir, dolutegravir, bictegravir, and cabotegravir. Higher docking scores, represented by more negative values, indicate stronger predicted binding affinities between the ligands and HIV-1 integrase (Table 2). These strong binding affinities predict the potential of these compounds to inhibit integrase-mediated processes in the HIV-replication cycle. Compounds 1, 6, 13, 3, and 7 consistently exhibited higher docking scores of −6.8, −6.6, −6.5, −6.2, and −5.9 kcal/mol, respectively, across HIV-1 integrase wild-type and resistant strains, suggesting that they may serve as a potent integrase inhibitor, even in resistant strains. On the other hand, hexamethylcyclotrisiloxane (Compound 10) and octamethylcyclotetrasiloxane (Compound 12) displayed the weakest binding, with scores of −3.2 kcal/mol and −3.4 kcal/mol, respectively. Their lower binding affinities suggest they may be less effective as integrase inhibitors. Furthermore, citraconic anhydride (Compound 5) and 2-Acetylcyclopentanone (Compound 2) with docking scores of −4.4 and −4.6 kcal/mol, respectively, also demonstrate weaker binding, potentially limiting their antiviral effectiveness. Nonetheless, the strong affinity of these compounds with integrase may produce the favourable antiviral activity of *A. alternata* crude extracts. Docking scores for the INSTIs show that bictegravir (−8.3, −8.5, and −8.2 kcal/mol) demonstrated slightly better binding affinity compared to raltegravir (−8.1, −8.4, and −8.2 kcal/mol), dolutegravir had comparable binding scores (−8.1, −8.3, and −8.1 kcal/mol), and cabotegravir had lower scores (−7.5, −7.6, and −7.6 kcal/mol) for wild type, T66K, and S230R, respectively.

### 2.11. Protein–Ligand Interaction Framework Analyses

**Figure 9:**
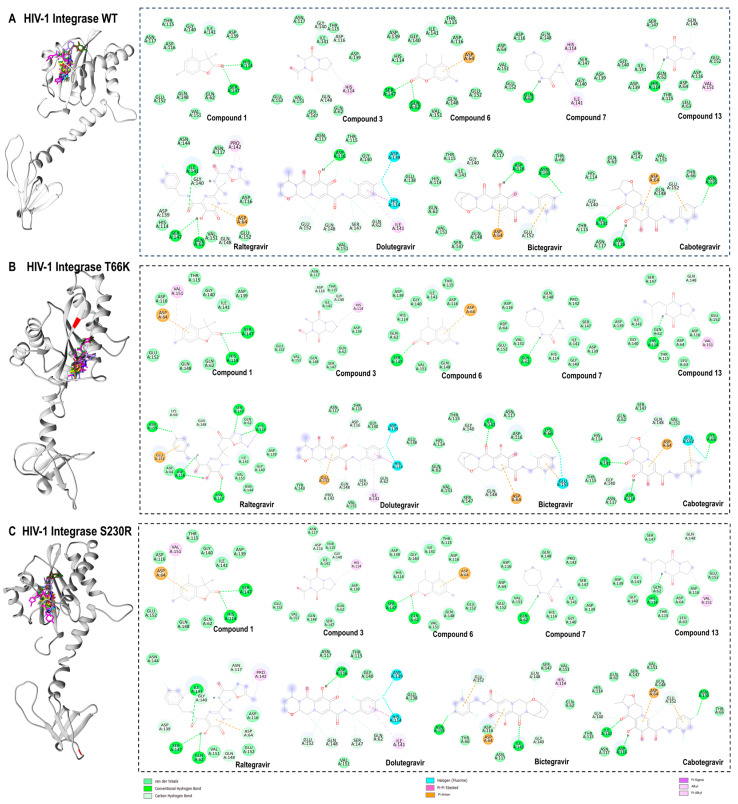
Three-dimensional (3D) docking pose of compound 1 (cyan), compound 3 (yellow), compound 6 (orange), compound 7 (blue), compound 13 (brown), raltegravir (magenta), dolutegravir (medium purple), bictegravir (forest green), and cabotegravir (brown) with HIV-1 integrase wild type (light grey) (**A**), T66K strain (grey) (**B**), S230R strain (dark grey) (**C**), and their respective two-dimensional (2D) protein–ligand interaction framework. Bond types are shown, and the topologies of T66K and S230R mutations are coloured red.

Compared to the reference compounds, the protein–ligand interactions of high-binding affinity compounds (compounds 1, 3, 6, 7, and 13) were analysed across HIV-1 integrase wild-type and mutant strains (T66K and S230R). By assessing these interactions, key differences and similarities in the binding modes were identified (Figure 9). Interactions of van der Waals were observed across all protein–ligand complexes with varying hydrogen bonds, attractive charges, and hydrophobic interactions.

There were several similarities and differences in how compounds interact with the HIV-1 integrase wild-type, T66K, and S230R strains (Figure 9A). For the wild-type integrase, compound 1 formed hydrogen bonds with HIS 114 and SER 147, whereas, in both the T66K and S230R strains, it retained similar interactions but also established additional pi–anion and alkyl interactions with ASP 64 and VAL 151, respectively. This suggests that the T66K and S230R mutations do not significantly alter the binding affinity of compound 1 compared to that of the wild type. Compound 3 demonstrated a consistent interaction pattern across all three strains, forming carbon–hydrogen bonds with ASP 116 and GLY 140 and a pi–alkyl interaction with HIS 114. This consistency suggests that the binding mode of compound 3 is relatively unaffected by mutations at positions T66K and S230R. Similarly, compound 6 formed hydrogen bonds with SER 147 and GLN 62 and a pi–anion interaction with ASP 64 in both the wild-type and S230R strains. However, in the T66K strain, it only formed a hydrogen bond with SER 147 and maintained the pi–anion interaction with ASP 64, indicating a slight variation in its binding dynamics due to the mutation. Compound 7 interacted with the wild-type integrase by forming a conventional hydrogen bond with GLN 62 and alkyl and pi–alkyl interactions with ILE 141 and HIS 114, respectively. This interaction profile was preserved in the T66K and S230R strains, except for the loss of hydrophobic interactions in these mutants. Compound 13 also showed consistent binding across all strains, forming hydrogen bonds with GLN 148 and HIS 114 and a pi–alkyl interaction, with an additional alkyl interaction involving VAL 151. This suggests that the binding mode of compound 13 is highly robust against mutations at T66K and S230R. Raltegravir, a known integrase strand transfer inhibitor, exhibited extensive and robust interactions across all strains. In the wild type, it forms numerous conventional hydrogen bonds, carbon–hydrogen bonds, and pi–alkyl interactions with various residues. In the T66K strain, raltegravir forms hydrogen bonds with LYS 66, ASN 155, ASP 116, ASN 117, GLN 148, SER 147, and HIS 114, and a pi–sigma interaction with HIS 114. This interaction pattern indicates high binding affinity and adaptability to the mutated residues. In the S230R strain, raltegravir maintained a similar interaction profile to the wild type, underscoring its effectiveness as an integrase inhibitor, even in the presence of mutations. Overall, the comparative analysis highlights that while certain compounds maintain consistent interaction profiles across all strains, others, such as compounds 6 and 7, show variations in binding affinity due to mutations.

In addition to raltegravir, the interactions of second-generation integrase inhibitors—dolutegravir, bictegravir, and cabotegravir—were examined across wild-type HIV-1 integrase and mutant strains (T66K and S230R) (Figure 9). These inhibitors demonstrated unique interaction patterns, showcasing their adaptability to integrase mutations and strong binding. Dolutegravir consistently formed fluorine halogen bonds with HIS 114 and ASP 139 across all strains. It retained its interactions in the wild-type and S230R strains but showed a slight shift in T66K, losing certain hydrogen bonds and forming a new carbon–hydrogen bond with PRO 142. Bictegravir demonstrated adaptability across strains, forming stable carbon and conventional hydrogen bonds with key residues like GLY 140, GLU 152, and ASP 116 in the wild type. In T66K, it introduced additional interactions, such as a fluorine halogen bond with GLU 152, and in S230R, it maintained robust binding through pi–alkyl and pi–anion interactions. Cabotegravir displayed a stable interaction profile across all strains, consistently forming hydrogen bonds with ILE 141, ASP 116, and ASN 155, along with pi–anion interactions with ASP 64 and GLU 152. The T66K mutation introduced a fluorine halogen bond with GLU 152, but the overall binding was preserved. Raltegravir and the second-generation integrase inhibitors maintained robust and versatile interaction profiles, showing their efficacy against various HIV-1 integrase strains.

## 3. Discussion

Measuring mitochondrial respiration was necessary to determine the cell viability and cytotoxicity of the *A. alternata* crude extract on the TZM-bl cell line. At greater concentrations of the *A. alternata* crude extract, the viability of TZM-bl cells decreased due to a decrease in mitochondrial respiration. The decrease in mitochondrial respiration was proportional to the reduction of MTT by mitochondrial succinate dehydrogenase in living cells [29]. The cell viability of TZM-bl cells treated with the *A. alternata* crude extract was greater than 80%. A 30% reduction in cell viability suggests a sample of interest has had a cytotoxic effect. Therefore, the *A. alternata* crude extract has not had a cytotoxic effect on TZM-bl cells [30]. A crude extract is said to have in vitro cytotoxicity if the CC_50_ of the extract is greater than 30–40 μg/mL [31]. Therefore, the *A. alternata* crude extract did not have a cytotoxic effect on the TZM-bl cell line. This finding agrees with the previous study that found *A. alternata* crude extract non-cytotoxic with a CC_50_ of 43.5 μg/mL [26].

The antiviral activity of the *A. alternata* crude extract on the PC 148 (subtype A), MC 2297 (subtype A), pNL4.3 (subtype B), YU2 (subtype B), CM7 (subtype C), CM9 (subtype C), PC 60 (subtype D), and PC 178 (subtype D) HIV-1 viruses were measured to determine inhibition as a percentage and the IC50. At higher and progressively lower concentrations, the *A. alternata* crude extract maintained antiviral activity greater than 50% inhibition. A decrease in antiviral activity was caused by a decrease in the concentration of the *A. alternata* crude extract, resulting in increased infection of TZM-bl cells by the respective viruses and, therefore, an increase in RLUs when measured. An extract can be described as highly active if the IC50 is less than 10 µg/mL, according to Moga et al. [32]. The *A. alternata* crude extract had an IC50 less than 10 µg/mL against the PC 148 (subtype A), MC 2297 (subtype A), pNL4.3 (subtype B), YU2 (subtype B), CM7 (subtype C), CM9 (subtype C), PC 60 (subtype D), and PC 178 (subtype D) viruses.

To date, the *A. alternata* crude extract has not been previously reported against PC 148, MC 2297, YU2, CM7, CM9, PC 60, and PC 178 viruses. The studies by Nzimande et al. [19] and Kubheka et al. [26] determined that the IC50 of the *A. alternata* crude extract against pNL4.3 ranges from 2.389 to 0.017 μg/mL. The *A. alternata* crude extract had an IC50 range of 0.1027 to 0.6086 μg/mL against the subtype A, B, C, and D viruses. The *A. alternata* crude extract was highly active against the subtype A, B, C, and D viruses. A sample with an SI value of 10 or greater can be said to have therapeutic potential and should be investigated further [33]. The SI values of the *A. alternata* crude extract against PC 148, MC 2297, pNL4.3, YU2, CM7, CM9, PC 60, and PC 178, as well as subtype A, B, C, and D viruses, were greater than 10 and can be said to have therapeutic potential. These results agree with those of Kubheka et al. [26], who found the *A. alternata* crude extract SI value of 2559 against the virus pNL4.3, which was greater than 10, indicating therapeutic potential. Values such as IC_50_ and SI can be useful predictors of drug usefulness clinically and have been used in previous antiretroviral drug studies such as allosteric integrase inhibitor 22 [34]. These values from in vitro studies are to be applied to in vivo studies such as clinical trials to further study drug efficacy.

The antiviral activity of the *A. alternata* crude extract, raltegravir, and dolutegravir was assessed on TZM-bl cells infected with integrase drug-resistant virus T66K and S230R utilizing the luciferase-based antiviral assay. The integrase drug-resistant virus T66K FC, in resistance to raltegravir and dolutegravir, was 13.15 and 1.864, respectively. It was previously reported that this mutation conferred an FC of 9.6 and 2 to raltegravir and dolutegravir, respectively [35,36]. The FC in susceptibility of integrase drug-resistant virus T66K was, therefore, similar to that previously reported by Kobayashi et al. [35] and Healthcare [36]. The integrase drug-resistant virus S230R had an FC in susceptibility to raltegravir and dolutegravir of 1.305 and 2.166, respectively. It was previously reported that this mutation conferred an FC in susceptibility of 1.52 and 3.85 to raltegravir and dolutegravir, respectively [37]. The FC in susceptibility of integrase drug-resistant virus S230R was, therefore, similar to that previously reported by Pham et al. [37].

The *A. alternata* crude extract had achieved 100–50% viral inhibition of integrase drug-resistant viruses T66K and S230R in decreasing concentrations from 300 μg/mL. The FC in susceptibility of integrase drug-resistant viruses T66K and S230R treated with *A. alternata* crude extract was 0.7265 and 0.8251, respectively. Since the FC in susceptibility was less than 1, it may suggest the integrase drug resistance was overcome, or there was another possible mechanism of action. The *A. alternata* crude extract had greater inhibitory activity than raltegravir against the integrase drug-resistant virus T66K. There was no remarkable difference in inhibition during integration when the integrase drug-resistant T66K virus was treated with the *A. alternata* crude extract and raltegravir. Raltegravir had a greater inhibitory effect than the *A. alternata* crude extract against the integrase drug-resistant virus S230R. The *A. alternata* crude extract and raltegravir had similar inhibitory during integration against integrase drug-resistant virus S230R. Raltegravir is a strand transfer inhibitor and, therefore, binds to integrase with greater affinity when the enzyme is in a complex with the viral DNA of HIV [38]. Raltegravir competes with DNA by binding to the DNA binding sites at residues such as N155 and Q148 [39]. Based on the inhibition profiles of the integrase drug-resistant strains, an alternate pathway, such as attachment and reverse transcription inhibition, could explain the activity of the *A. alternata* crude extract against the integrase drug-resistant mutants. However, to validate the integrase activity of the *A. alternata* crude extract during the time of addition, integrase activity assays and Alu gag PCR are recommended to determine the integrase inhibitory activity of the crude extract.

The mechanism of action of the *A. alternata* crude extract was assessed by luciferase-based and HIV-1 p24-based methods on TZM-bl and Jurkat cells. The time-of-addition assay based on the luciferase method revealed that the *A. alternata* crude extract had activity during attachment, reverse transcription, and integration among the viruses pNL4.3, YU2, CM9, CM7, PC60, MC2297, PC148, PC178, and T66K. In addition, *A. alternata* crude extract had activity during attachment, reverse transcription, and integration activity when CM9, MC 2297, and S230R were treated. The HIV-1 p24-based method revealed that the *A. alternata* crude extract had activity during attachment, reverse transcription, and integration when the viruses CM9, MC2297, and T66K were treated. In addition, the *A. alternata* crude extract had activity during attachment, reverse transcription, and integration activity when the virus S230R was treated. The *A. alternata* crude extracts have many secondary metabolites that may have different antiviral activity against HIV-1. Isolation of these secondary metabolites may show the specificity of each compound in HIV-1 replication. Therefore, the *A.alternata* crude extracts may contain the compounds that inhibit HIV-1 replication in different stages.

Based on the results above, it is evident that the *A. alternata* crude extract has activity during attachment, reverse transcription, and integration. However, activity during proteolysis appears to be weaker. Identifying and isolating the compounds that may have anti-HIV-1 activity during these stages is essential. Kubheka et al. [26] showed that the *A. alternata* crude extract had a similar activity profile during attachment, reverse transcription, and integration. However, it demonstrated greater activity during proteolysis using both luciferase and p24-based time-of-addition methods against the pNL4.3 virus. In agreement with our finding, the fractionated *A. alternata* crude extract demonstrated an even greater activity during attachment, reverse transcription, integration, and proteolysis [26]. This may indicate that an active compound is captured in the MCX fraction of the *A. alternata* crude extract, which may explain the increased activity. The antiviral compounds cyclotrisiloxane octamethyl, propionitrile, pyrrolol[1,2-a]pyrazine-1,4-dione, hexahydro-3-(2-methyl propyl) (10.42%), silane, diethyl ethoxy (2-ethoxyethyloxy), coumarin, 3,4-dihydro-4,5,7-trimethyl-4,5,7-trimethyl2-chromanone, and 1,2-cyclobutanedicarbonitrile partially identified by GC-MS could be responsible for these activities described above [19]. The coumarin class of compounds has previously been demonstrated to inhibit reverse transcriptase, integrase, and protease inhibitory activity of 82.81%, 98%, and protease 78%, respectively [24]. The presence of coumarins potentially may explain the time-of-addition profile described in this study.

Docking studies demonstrated that all 14 identified compounds have varying binding affinity to HIV-1 integrase wild-type and T66K/S230R-resistant strains. Notably, the antiviral compounds 2,3-2H-Benzofuran-2-one, 3,3,4,6-tetramethyl-, 3-Methyl-1,4-diazabicyclo[4.3.0]nonan-2,5-dione, N-acetyl, Coumarin, 3,4-dihydro-4,5,7-trimethyl-, Cyclopropanecarboxamide, N-cycloheptyl, Pyrrolo[1,2-a]pyrazine-1,4-dione, and hexahydro-3-(2-methylpropyl)- exhibited particularly strong binding, as indicated by their high docking scores. The robust antiviral effects of these compounds are likely due to the intricate synergy of van der Waals forces, hydrogen bonds, and hydrophobic interactions, which contribute to their stability and specificity in inhibiting these viral targets, as reported in this study. These interactions enhance the compounds’ binding affinity and support their potential as effective antiviral agents against HIV-1 integrase wild-type and resistant strains. For example, the maintenance of stable hydrogen bonds by compound 1 with HIS 114 and SER 147 while forming additional hydrophobic interactions in the mutant strains highlights its adaptability to structural changes. Compound 3 maintained consistent carbon–hydrogen bonds with ASP 116 and GLY 140 across all strains, demonstrating its resilience to mutations. Compound 6 retained hydrogen and hydrophobic interactions with SER 147 and ASP 64, respectively, although the loss of the hydrogen bond with GLN 62 was observed in the T66K strain. Compound 7 formed hydrophobic contacts with ILE 141 and HIS 114 in the wild-type strain, which was lost in mutant strains, even though the hydrogen bond with GLN 62 was preserved across all strains. Compound 13 exhibited robust binding across all strains, forming hydrogen bonds with GLN 148 and HIS 114, along with hydrophobic interaction with VAL 151. These findings underscore the crucial role of hydrogen bonding and hydrophobic interactions in stabilizing the binding of these compounds, even in the presence of mutations, and support the mechanistic basis for the predicted inhibitory affinities. Furthermore, the robust interaction profiles of reference integrase inhibitors not only underscore their effectiveness against resistant HIV-1 strains but also reinforce their value in combination therapy regimens.

## 4. Materials and Methods

### 4.1. Extraction of Crude Alternaria alternata Secondary Metabolites

Fungal plugs (6 mm^2^) of *Alternaria alternata*, PO4PR2, were isolated from Hypoxis species (voucher no. 18233) on 5-day-old malt agar and identified by Nzimande et al. [19]. The isolated *A. alternata*, PO4PR2, was inoculated into a 1-litre Erlenmeyer flask (Rankem, Haryana, India) containing 200 mL of malt extract broth and incubated for 21 days at 30 °C under static conditions in the dark. The fungal cultures were then extracted with 200 mL of methanol (Sigma–Aldrich, Johannesburg, South Africa) by shaking at 150 rpm using an Eins-Sci^®^ benchtop Rotary shaker (Reflecta Laboratory supplies, Germiston, South Africa). The fungal cultures were then filtered using sterile gauze to separate the mycelia from the methanol extract and dried at 40 °C under a mild nitrogen gas steam. The dried *A. alternata*, PO4PR2, crude extract was kept at −80 °C and diluted to a working concentration of 300 µg/mL before using 0.2% DMSO (dimethyl sulphoxide).

### 4.2. Maintenance of Cell Lines

The TZM-bl and 293T/17 cell lines (NIH AIDS Research and Reference Reagents Prgramme) were cultured as a monolayer in sterile 75 cm2 culture flasks using Dulbecco’s Modified Eagle Medium (DMEM) (Thermo Fisher Scientific, Waltham, MA, USA) supplemented with 10% fetal bovine serum (FBS; heat-inactivated and gamma irradiated) (LTC Biosciences, Gainesville, FL, USA), 25 mM of HEPES (Thermo Fisher Scientific, Waltham, MA, USA), and 50 µL/mL of gentamicin (Thermo Fisher Scientific, Waltham, MA, USA). The TZM-bl cell line is modified from a HeLa cell line clone to express CD4 and CCR5, allowing for HIV-1 infection and firefly luciferase under the control of the HIV-1 long-terminal repeat (LTR) [40]. The 293T/17 cell line is a highly transfectable cell line that produces high titres of HIV-1 virions [41]. The TZM-bl and 293T/17 cell lines were incubated at 37 °C with 5% CO_2_ to confluency. At confluency cells were washed with phosphate-buffered saline (PBS) (Thermo Fisher Scientific, Waltham, MA, USA). Cell monolayers were then trypsinised with 0.25% Trypsin-EDTA (Thermo Fisher Scientific, Waltham, MA, USA). Trypsinized cells were then counted using 0.4% trypan blue dye and subcultured until confluency was reached. The Jurkat cell line, which is an immortalized cell line of human T lymphocyte cells, was maintained in RPMI-1640 media (Thermo Fisher Scientific, Waltham, MA, USA), supplemented with 10% (*v*/*v*) heat-inactivated fetal bovine serum (FBS), and incubated at 37 °C with 5% CO_2._

### 4.3. Cytotoxicity and Cell Viability Testing of A. alternata Crude Extract Using MTT Assay (3-[4,5-Dimethylthiazol-2-yl]-2,5 Diphenyl Tetrazolium Bromide)

*A. alternata* crude extract cell cytotoxicity and viability were assessed by MTT (3-[4, 5-dimethylthiazol-2-yl]-2, 5-diphenyltetrazolium bromide) cell viability assay using the CYQUANT MTT cell proliferation kit (Thermo Fisher Scientific, Waltham, MA, USA). A 96-well culture plate was seeded with 15,000 TZM-bl cells/200 µL of DMEM per well and incubated at 37 °C with 5% CO_2_.). Following this, 10 µL of the drug control azidothymidine (NIH AIDS Research and Reference Reagent Programme) and the *A. alternata* crude extract were diluted five-fold, and control wells where neither the control drug AZT nor the crude extract was added were followed by incubation at 37 °C with 5% CO_2_ and 95% humidity for 48 h. Ten microlitres of 12 mM of MTT solution was added to each well and incubated for 4 h at 37 °C with 5% CO_2_ and 95% humidity. Then, 50 µL of 100% DMSO were added to each well and incubated for 10 min. The absorbance was then measured at a wavelength of 540 nm using the Victor Nivo microplate reader (PerkinElmer, Waltham, MA, USA). The percentage of cell viability was calculated using the formula below [18]:

%Cell viability = (Sample absorbance − media control)/(Mean media control absorbance) × 100

The drug control azidothymidine and *A. alternata* crude extract CC_50_ (50% cytotoxic concentration) were determined using GraphPad Prism software (v.5) by dose-response curve.

### 4.4. Plasmid DNA Preparation and Purification

The glycerol stocks of the plasmids pNL4.3 (subtype B), YU2 (subtype B), CM7 (subtype C), and CM9 (subtype C) were cultured in 250 mL of prewarmed LB (Luria–Bertani) broth supplemented with 1% ampicillin (Sigma-Aldrich, St. Louis, MO, USA) at 37 °C in a shaking incubator at 230 RPMI. Plasmid DNA was then prepared and purified using the QIAGEN Plasmid Maxi Kit (QIAGEN, Hulsterweg, The Netherlands) following the manufacturer’s instructions. The Nanodrop OneTM and 1% gel electrophoresis assessed plasmid DNA quality and purity.

### 4.5. Site-Directed Mutagenesis

#### 4.5.1. Design of Site-Directed Mutagenesis Primers

The Stanford University HIV drug-resistance database was analysed for drug-resistance mutations that confer resistance to raltegravir and dolutegravir [42]. Integrase mutations were selected that conferred resistance to raltegravir and dolutegravir as single mutations and caused a fold change in susceptibility to raltegravir and dolutegravir greater than 1. Integrase inhibitor drug-resistance mutations T66K, E92Q S230R, and R263K were then selected based on the parameters described above. The primers in Table 3 were then designed to introduce the respective mutations T66K, E92Q, S230R, and R263K using the NEBaseChanger primer design tool (New England Biolabs, Massachusetts, United States of America). The Q5 site-directed mutagenesis kit (Ipswich, Massachusetts, United States of America) was used to introduce the respective T66K, E92Q, S230R, and R263K mutations into HIV-1 molecular clone pNL4.3 using the primers designed.

#### 4.5.2. Site-Directed Mutagenesis PCR Reaction

Following the manufacturer instructions of the Q5 site-directed mutagenesis kit, an exponential amplification polymerase chain reaction (PCR) was conducted for each respective mutation (New England Biolabs, Ipswich, MA, USA). The plasmid pNL4.3 was used as the template for site-directed mutagenesis since it is an HIV-1 recombinant viral clone of HIV-1. Further, 12.5 µL of Q5 hot-start high-fidelity master mix (Ipswich, MA, USA) were administered into a thin-walled PCR tube, as well as 1 µL at 25 ng/µL of pNL4.3 DNA template, 9 µL of deionised water, and 1 µL–10 µM of the respective forward and reverse primers: T66K_FWD, T66K_REV, E92Q_FWD, E92Q_REV, S230R_RWD, S230R_REV, R263K_FWD, and R263K_REV. The 25 µL PCR reaction was mixed and thermocycled on the Applied Biosystems miniamp thermal cycler (Thermo Fisher Scientific, Waltham, MA, USA) using the cycling conditions 98 °C for 30 s, 98 °C for 10 s, 58–61 °C for 7 min 15 s, and 25 cycles and 72 °C for 2 min. Parental DNA was then digested using DpnI, and PCR products were transformed into competent Escherichia coli cells. Plasmid DNA was then extracted and purified using the GeneJET miniprep kit following the manufacturer’s instructions (Waltham, MA, USA).

### 4.6. Sequencing

#### 4.6.1. Integrase PCR Amplification

The HotStarTaq Master Mix (QIAGEN, Hulsterweg, The Netherlands) was used to PCR amplify the integrase gene of the selected site-directed mutants. The PCR reaction was assembled according to the manufacturer’s instructions. In a thin-walled PCR tube, 25 μL of HotStarTaq, 1 μL (0.5 μM) of forward primer 4155F- GTACCAGCACACAAAGGRATTG, 1 μL (0.5 μM) of reverse primer 5264R-CCTGTATGCAGACCCCAATATGTT, 1 μL (<1 μg) of DNA template, and 22 μL of RNAse-free water were added and mixed [43]. The reaction mixture was then placed in the Applied Biosystems miniamp thermal cycler (Thermo Fisher Scientific, Waltham, MA, USA) using the conditions 95 °C for 5 min and 1 cycle, 94 °C for 1 min, 55 °C for 1 min, 72 °C for 3 min, 30 s for 30 cycles, and 72 °C for 10 min and 1 cycle. The integrase PCR amplicons were confirmed and assessed using the Nanodrop OneTM and 1% gel electrophoresis.

#### 4.6.2. Cycle Sequencing Reaction

The BigDye Terminator v3.1 cycle sequencing kit (Thermo Fisher Scientific, Waltham, MA, USA) master mix reaction was prepared using 2 µL of v3.1 5X Sequencing Buffer, 3.2 µL and 10 µM of the respective forward and reverse primers 4155F and 5264R, 0.4 µL of BigDye Terminator v3.1 Ready Reaction Mix, and 20 ng of DNA template, and the reaction volume was filled up to 10 µL with deionised water. Then, 10 µL of the reaction mix were aliquoted into a MicroAmpTM Optical 96-well reaction PCR plate (Thermo Fischer Scientific, Waltham, MA, USA) and mixed. The positive control from the BigDye Terminator v3.1 cycle sequencing kit was set up using the forward primer M13-TGTAAAACGACGGCCAGT and PGEM as the template. The MicroAmpTM Optical 96-well reaction PCR plate was then thermocycled using the parameters 96 °C for 1 min, 96 °C for 10 s, 50 °C for 5 s, 60 °C for 4 min, and 25 cycles kept on hold for 4 °C indefinitely (Applied Biosystems miniamp thermal cycler, Thermo Fischer Scientific, Waltham, MA, USA). The products were then sequenced by capillary electrophoresis on the 3500 Genetic analyser (Applied Biosystems, Waltham, MA, USA). The full-length integrase gene sequences were then assembled and aligned in Unipro UGENE (v.50.0, Unipro) using the contig assembly program version 3 (CAP3).

### 4.7. Generation of Different HIV-1 Subtypes by Transfection

To prepare the HIV-1 subtypes, prepared HEK 293T cells were transfected with the relevant HIV-1 virus using the plasmid stocks generated by plasmid DNA preparation and purification of (pNL4.3, pYU2, pCM7, and pCM9) plasmids kindly donated by Dr. Omolara Baiyegunhi [44]. Briefly, 3 × 106 HEK 293T cells in the T-75 culture flask were seeded and incubated overnight at 37 °C with 5% CO_2_ and 95% humidity. Plasmid DNA from transfected HEK 293T cells in T-75 cell culture flasks using Fugene transfection reagent (Promega, Fitchburg, WI, USA). After 4 h, the transfected HEK 293T cells’ medium was changed, and they were then incubated for 48 h at 37 °C, 5% CO_2_, and 95% humidity. The supernatant of the transfected cells was then harvested and filtered with a 0.45 µm filter aliquoted in 2 mL cryovials and stored at −80 °C.

### 4.8. Titration of HIV-1 Viruses

The infectivity of the generated HIV-1 viruses was evaluated by infecting TZM-bl cell lines. The viruses’ TCID50 (50% tissue culture infectious dose) was determined. Then, 100 µL of DMEM supplemented with 10% FBS, 25 mM of HEPES buffer, and 1% penicillin–streptomycin (Pen Strep) were added to a 96-well cell culture plate. In replicates of 4, 25 µL of the virus stock produced by transfection were added to four wells in row 1 and serially diluted four-fold. Then, 10,000 TZM-bl cells containing 37.5 µg/mL DEAE-dextran (Diethylaminoethyl-dextran) to allow for infection were added and incubated for 48 h at 37 °C with 5% CO_2_ and 95% humidity. From each well, 100 µL of cell culture medium was removed, and in the absence of light, 100 µL of Bright gloTM luciferase reagent were added, except for the control in row 12. The supernatant was aspirated in each well, 150 µL were transferred to the black 96-well plate, and the RLUs (relative light units) were immediately quantified in a luminometer at 540 nm (PerkinElmer, Shelton, CT, USA). The TCID50 was extrapolated in the plotted graph by selecting the dilution concentration that elicited a minimum of 15,000 and a maximum of 50,000 relative light units (RLU).

### 4.9. Luciferase-Based Antiviral Assay

An antiviral assay based on luciferase assessed the HIV-1 inhibition of fungal extracts [19,26,45]. The luciferase-based assay was conducted using the viruses generated by transfection. Firstly, 150 µL, 100 µL, and 140 µL of DMEM were added to a 96-well cell culture plate as a cell control, virus control, and sample, respectively. Briefly, 11 µL of the drug controls (azidothymidine or raltegravir and dolutegravir) and *A. alternata* crude extract were added to a Corning Costar 96- well cell culture plate (Corning, New York, NY, USA), and three-fold dilution was performed in 140 µL of DMEM supplemented with 10% FBS, 25 mM of HEPES buffer, and 1% penicillin–streptomycin. Infected TZM-bl cell lines (virus control) and uninfected TZM-bl cell lines (cell control) were included as the experimental control. Then, 50 µL of PC148, MC2297, pNL4-3, YU2, CM7, CM9, PC60, PC178, T66K, and S230R viruses at TCID50 were added to the 96-well cell culture plate and incubated for 1 h. In each 96-well cell culture plate well, 10,000 cells were added and cultured for 48 h at 37 °C with 5% CO_2_ and 95% humidity with 37.5 µg/mL of DEAE-dextran. After 48 h of incubation, 150 µL of media were removed and replaced with 100 µL of Bright–Glo TM luciferase reagent without light exposure. The supernatant was aspirated and 150 µL of the supernatant and Bright–Glo TM luciferase reagent mixture were added to a Corning Costar 96-well black plate. It was immediately measured in the Victor Nivo microplate reader at 540 nm (PerkinElmer, Waltham, United States of America). Then, the percentage of viral inhibition was calculated using the formula below:

% HIV inhibition = (average sample − average control)/(1 − (average viral control − average control)) × 100

The IC50 (inhibitory concentration) was then determined by a dose-response curve using GraphPad Prism software (v.5).

### 4.10. Time-of-Addition Assay-Based Inhibitory Mechanism in the HIV-1 Life Cycle

#### 4.10.1. Luciferase-Based Time-of-Addition Assay

The time-of-addition assay was used to determine how the anti-HIV drugs interfered with the HIV-1-replication cycle. The luciferase-based time of addition was conducted as described by Lara et al. [46] and Kubheka et al. [26]. Briefly, in a 96-well cell culture plate, 150 µL of 10 000 TZM-bl cells were seeded in each well and incubated at 37 °C, 5% CO_2_. The TZM-bl cells were then infected with 50 µL of each HIV-1 subtype (PC148, MC2297, pNL4-3, YU2, CM7, CM9, PC60, and PC178) and T66K and S230R INSTI drug-resistant strains. Then, 15 µL of antiretroviral drug controls maraviroc (CCR5 antagonist, 1 h), azidothymidine (5 h), raltegravir (8 and 10 h), and amprenavir (16 h) were added to a well-contained infected cell. The *A. alternata* crude extract was added at 1, 3, 5, 6, 8, 10, 16, and 20 h post-infection. The 96-well cell culture plate was then incubated at 37 °C with 5% CO_2_ for 48 h. Then, 150 µL of cell culture medium were removed and 100 µL of Bright–Glo luciferase reagent (Promega, Madison, WI, USA) were added and mixed. Then, 150 µL of the Bright–Glo luciferase reagent (Promega, Madison, WI, USA) and cell medium mixture were transferred to a 96-well black plate and measured at 540 nm in luminometer (Promega, Madison, WI, USA). The percentage inhibition at each time point was calculated using the formula below.

% HIV inhibition = (average sample − average control)/(1 − (average viral control − average control)) × 100

#### 4.10.2. HIV-1 p24 Time-Based ELISA to Measure p24 Titre

The HIV-1 p24-based time-of-addition assay was performed using a modified protocol [47,48]. The Jurkat cell line was maintained in RPMI-1640 media (Gibco, Thermo Fisher Scientific, Waltham, MA, USA) supplemented with 10% (*v*/*v*) heat-inactivated fetal bovine serum (FBS) and incubated at 37 °C with 5% CO_2_. To a Corning Costar 24-well cell culture plate (Corning, USA, New York), 500 µL of 500,000 Jurkat cells were maintained under 37 °C, and 5% CO_2_ was seeded in each well. Then, all wells with Jurkat cells were infected with 100 µL of HIV-1 viruses (CM9, MC2297) and integrase drug-resistant strains T66K and S230R. The CM9 and MC2297 viruses were selected since the *A. alternata* crude extract had the most potent anti-HIV activity against these two viruses. The integrase drug-resistant strains T66K and S230R were selected since these were the only functional integrase-resistant strains available. Then, 50 µL of antiretroviral drug controls maraviroc (CCR5 antagonist, 0–1 h), azidothymidine (nucleoside reverse transcriptase inhibitor 3 h), raltegravir (INSTI inhibitor 8–14 h), and amprenavir (protease inhibitor 14–24 h) were added to well-contained infected cells. The *A. alternata* crude extract was added to infected cells at 0, 1, 3, 8, 10, 14, 24, and 30 h post-infection. After 48 h post-infection, the HIV-1 p24 titre was measured using the Quicktiter Lentiviral Quantification kit (Cell Biolabs Inc., San Diego, United States of America) following the manufacturer’s instructions. The HIV-1 p24 was then measured at an absorbance of 450 nm using the Victor Nivo microplate reader (Promega, Madison, Wisconsin, United States of America).

The viral titre of p24 was calculated following manufacturer’s instructions below: The average genome size of lentivirus is 8 kbp; therefore, 1 ng of lentiviral RNA = (1 × 10^−9^) g/(8000 bp × 660 g/bp) × 6 × 1023 = 1.1 × 108 VP; virus titre = (amount of lentiviral RNA (ng) × 1.1 × 108 VP × (20 µL/5 µL))/(viral sample volume (mL)); virus titre (VP/mL) = (amount of lentiviral RNA (ng) × 4.4 × 108 VP/ng)/(viral sample volume (mL)).

### 4.11. Computational Methodology

#### 4.11.1. Preparation and Validation of Protein Structures

Molecular modeling studies focused on HIV-1 integrase protein owing to several factors. First, the time-of-addition assay showed strong inhibition during integration. Secondly, integrase inhibitors are still considered to be a class of antiretrovirals that have been developed recently. Moreover, integrase inhibitors are included in first-line regimens that are prescribed for people living with HIV/AIDS in South Africa and in many other countries.

The crystallized structure of wild-type HIV-1 integrase (PDB: 1EX4) was obtained from the RCSB Protein Data Bank https://www.rcsb.org/ (accessed on 15 September 2024). The structure was resolved via X-ray diffraction at high resolution of 2.8 Å [49]. Missing residues in the flexible loop region (amino acids 142–144) of the integrase protein were modeled using the MODELLER program [50]. The best model was selected based on the lowest zDOPE score to ensure optimal structural accuracy and reliability. Subsequently, point mutations of residues T66 and S230 to K and R were introduced using the Dunbrack Rotamer Library 2010 to refine the model further [51]. The active binding sites of the target protein were mapped by identifying the interacting residues of the crystalised ligand (raltegravir), providing a comprehensive understanding of the interaction landscape essential for docking calculations [52].

#### 4.11.2. Preparation of Ligand Structures

Chemical SMILES of the bioactive compounds (Figure S3) were retrieved from PubChem https://pubchem.ncbi.nlm.nih.gov/ (accessed on 25 September 2024), and their structures were generated using MarvinSketch 24.3.0 https://chemaxon.com/marvin (accessed on 16 September 2024) (Figure S2). The ligands were first optimized by minimizing their energies with the steepest descent algorithm and the GAFF force field in Avogadro 1.2.0 [53]. Additionally, energy minimization was performed using UCSF Chimera Tools, employing 100 steps of the steepest descent with a step size of 0.02 Å, followed by 10 steps of conjugate gradient minimization with a step size of 0.02 Å, updated at 10 intervals [54]. This rigorous preparation ensured that the ligands were in their most stable conformations for docking calculations.

#### 4.11.3. Molecular Docking Calculations

Docking calculations were performed using AutoDock Vina 1.1.2, employing the Lamarckian Genetic Algorithm [55]. AutoDock Tools was utilized to define grid maps based on the predicted active sites of the target proteins. The grid center coordinates were set at specific points for HIV-1 integrase (wild-type/T66K/S230R) (−10.255 Å, 41.169 Å, −40.213 Å) along the x, y, and z dimensions, respectively. The grid map size was set to 20.018 Å, 21.889 Å, and 20.019 Å along the x, y, and z dimensions, respectively. AutoDock Vina employs an advanced gradient optimization method during its local optimization process, which leverages directional information from a single evaluation to enhance the accuracy of the docking results. Docking poses with the lowest binding energies were selected and visualized in UCSF Chimera 1.18 for subsequent analysis, providing a foundation for understanding the molecular interactions between the target proteins and ligands. While AutoDock may have limitations in predicting interactions within flexible binding sites, it has been validated against experimental data through various benchmarking studies. One common approach is the redocking test, where known experimental ligands are re-docked into their respective receptors, as demonstrated in our previous study [54].

Due to the lack of available crystal structure for the HIV-1 integrase protein bound to viral DNA, which is critical for optimal INSTI binding, the integrase protein structure alone was utilised for the docking study. Raltegravir, a first-generation INSTI, was selected as a reference compound due to its specificity for the catalytic core of HIV integrase, allowing for a foundational comparison of binding affinities and interaction profiles. To provide a more comprehensive overview, the study also incorporated cabotegravir—a long-acting injectable INSTI—and second-generation INSTIs, dolutegravir and bictegravir, which have demonstrated efficacy against integrase mutations such as T66K and S230R.

#### 4.11.4. Protein–Ligand Interaction Assessment

The binding modes of the ligands and their interactions with the protein targets were visualized using Biovia Discovery Studio Visualizer 2024 [54]. This software enabled a detailed examination of non-covalent interactions, such as hydrogen bonds, hydrophobic interactions, van der Waals forces, and electrostatic interactions within each protein–ligand complex. The binding patterns were analysed and compared with those of the reference ligand, allowing for a comprehensive understanding of how the identified compounds interact with the protein targets.

## 5. Conclusions

The *A. alternata* crude extract demonstrated strong antiviral activity against HIV subtype A, B, C, and D viruses and integrase-resistant strains T66K and S230R. The findings of this study also showed that the crude extract has promising therapeutic potential and inhibits stages of the HIV life cycle. The in silico results showed that bioactive compounds from the crude extract had high affinities and strong binding interactions with HIV integrase. We can, therefore, conclude that the *A. alternata* crude extract is a promising source of anti-HIV compounds. Our results will further investigate and recommend that the bioactive compounds responsible for the anti-HIV activity in the *A. alternata* crude extract be isolated to target multiple stages of HIV replication.

## Figures and Tables

**Table 1:** Half-maximal inhibitory concentrations (IC50) and selectivity indices (SI) of *A. alternata* crude extract and drug control azidothymidine against different HIV-1 subtypes.

Virus	Subtype	IC_50_ µg/mL (Crude Extract)	IC_50_ µg/mL (Azidothymidine)	SI (Crude Extract)	SI (Azidothymidine)
PC 148	A	0.1650 ± 0.035	0.04025 ± 0.002	1818	9565
MC 2297	A	0.1029 ± 0.024	0.01383 ± 0.001	2915	27,838
pNL4.3	B	0.2383 ± 0.015	0.03377 ± 0.004	1259	11,401
YU2	B	0.3821 ± 0.056	0.04226 ± 0.004	785	9110
CM7	C	0.1739 ± 0.032	0.05798 ± 0.009	1725	6640
CM9	C	0.1027 ± 0.019	0.09241 ± 0.007	2921	37,487
PC 60	D	0.3796 ± 0.037	0.07795 ± 0.005	790	4939
PC 178	D	0.6086 ± 0.025	0.08129 ± 0.003	493	4736

**Table 2:** Docking scores of the identified secondary metabolites from *A. alternata* with bioactive and anti-HIV properties against HIV-1 Integrase wild-type and T66K- and S230R-resistant strains compared with raltegravir.

ID	Compound Name	Docking Score (kcal/mol)		
		HIV-1 Integrase Wild Type	HIV-1 Integrase T66K	HIV-1 Integrase S230R
1	2,3-2H-Benzofuran-2-one, 3,3,4,6-tetramethyl-	−6.8	−6.8	−6.8
2	2-Acetylcyclopentanone	−4.6	−4.6	−4.6
3	3-Methyl-1,4-diazabicyclo[4.3.0]nonan-2,5-dione, N-acetyl	−6.2	−6.2	−6.2
4	Asarone	−5.7	−5.6	−5.6
5	Citraconic anhydride	−4.4	−4.4	−4.4
6	Coumarin, 3,4-dihydro-4,5,7-trimethyl-	−6.6	−6.6	−6.6
7	Cyclopropanecarboxamide, N-cycloheptyl	−5.9	−5.9	−5.9
8	Ethyl phenylpropiolate	−5.5	−5.5	−5.6
9	Hexahydropyrrolo[1,2-a]pyrazine-1,4-dione	−5.7	−5.7	−5.7
10	Hexamethylcyclotrisiloxane	−3.2	−3.2	−3.2
11	Levoglucosenone	−4.6	−4.6	−4.6
12	Octamethylcyclotetrasiloxane	−3.4	−3.4	−3.4
13	Pyrrolo[1,2-a]pyrazine-1,4-dione, hexahydro-3-(2-methylpropyl)-	−6.5	−6.5	−6.5
14	Pyrrolo[1,2-a]pyrazine-1,4-dione, hexahydro-	−5.5	−5.5	−5.5
	Raltegravir	−8.1	−8.4	−8.2
	Dolutegravir	−7.5	−7.6	−7.6
	Bictegravir	−8.3	−8.5	−8.2
	Cabotegravir	−8.1	−8.3	−8.1

**Table 3:** Site-directed mutagenesis primers were designed using NEBaseChanger primer design tool to introduce the respective mutations T66K, E92Q, S230R, and R263K.

Primer Name	Sequence (5′-3′)
T66K_FWD	ATTAGATTGTAAACATTTAGAAGGAAAGA
T66K_REV	TGCCATATCCCTGGACTAC
E92Q_FWD	TATCCCAGCACAAACAGGACAAG
E92Q_REV	ACCTCTGCCTCTATATATCC
S230R_RWD	TTACAGAGACCGCAGAAATCCAC
S230R_REV	TAAACCCGAAAATTTTGAATTTTTG
R263K_FWD	AGTACCAAGGAAAAAAGTAAAAATC
R263K_REV	ACCTTTATGTCACTGTTATCTTGTATTAC

## Data Availability

Data is contained within the article or Supplementary Material.

## Supplementary Materials

The following supporting information can be downloaded at https://www.mdpi.com/article/10.3390/ph18020189/s1, Figure S1: Sequence alignment of integrase T66K (A), E92Q (B), S230R (C), and R263K (D) site-directed mutants with wild-type integrase gene. Figure S2: Two-dimensional (2D) chemical structures of the identified secondary metabolites of *A. alternate*. Figure S3: Gas chromatography–mass spectrometry (GC-MS) reports of compounds. Table S1: Secondary metabolites from *A. alternata* with bioactive and anti-HIV properties identified by GC-MS.
