# Supplementary material for: Alternaria alternata (Fr) Keissl Crude Extract Inhibits HIV Subtypes and Integrase Drug-Resistant Strains at Different Stages of HIV Replication
Source: Pharmaceuticals (Basel). 2025 Jan 30;18(2):189. doi: 10.3390/ph18020189 (PMC11859181; doi:10.3390/ph18020189)
Supplement: Supplementary file 1 [file pharmaceuticals-18-00189-s001.zip › pharmaceuticals-3434300-supplementary.pdf]

Table S1: Secondary metabolites from *A. alternata* with bioactive and anti-HIV properties identified by GC-MS

**Formula**	**Name (IUPAC)**	**Similarity index**
$C_7H_{10}N_2O_2$	Hexahydropyrrolo[1,2-a]pyrazine-1,4-dione	92
$C_8H_{24}O_4Si_4$	Octamethylcyclotetrasiloxane	89
$C_6H_6O_3$	Levoglucosenone	92
$C_{11}H_{10}O_2$	Ethyl phenylpropiolate	70
$C_{10}H_{14}N_2O_3$	3-Methyl-1,4-diazabicyclo[4.3.0]nonan-2,5-dione, N-acetyl	83
$C_{12}H_{16}O_3$	Asarone	38
$C_6H_{18}O_3Si_3$	Hexamethylcyclotrisiloxane	67
$C_{12}H_{14}O_2$	Coumarin, 3,4-dihydro-4,5,7-trimethyl-	56

C7H10N2O2	Pyrrolo[1,2-a]pyrazine-1,4-dione, hexahydro-	92
C5H4O3	Citraconic anhydride	69
C11H18N2O2	Pyrrolo[1,2-a]pyrazine-1,4-dione, hexahydro-3-(2-methylpropyl)-	76
C11H19NO	Cyclopropanecarboxamide, N-cycloheptyl	59
C12H14O2	2,3-2H-Benzofuran-2-one, 3,3,4,6-tetramethyl-	53

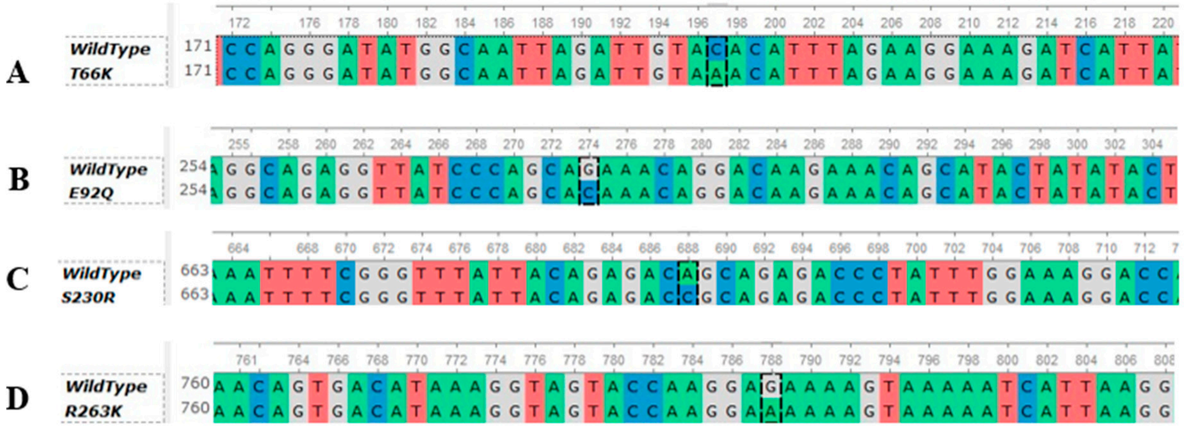

Figure S1: Sequence alignment of integrase T66K **(A)**, E92Q **(B)**, S230R **(C)** and R263K **(D)** site-directed mutants with wildtype integrase gene.

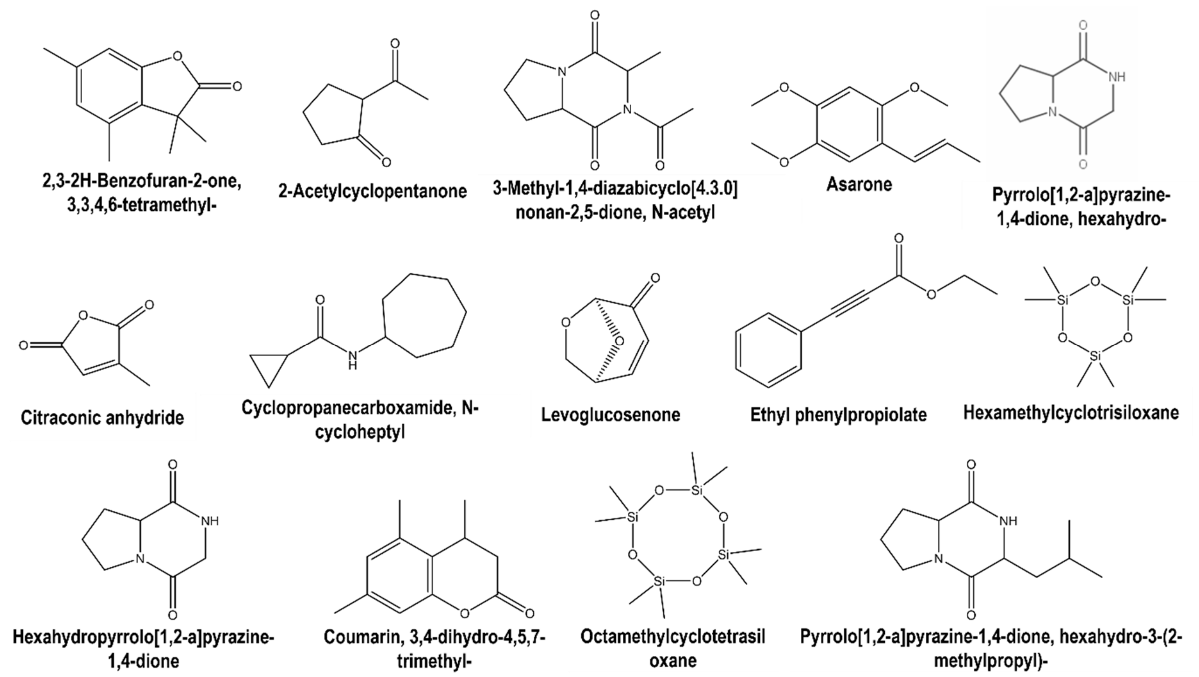

Figure S2: Two-dimensional (2D) chemical structures of the the identified secondary metabolites of *A. alternata* (Nzimande *et al*., 2022).

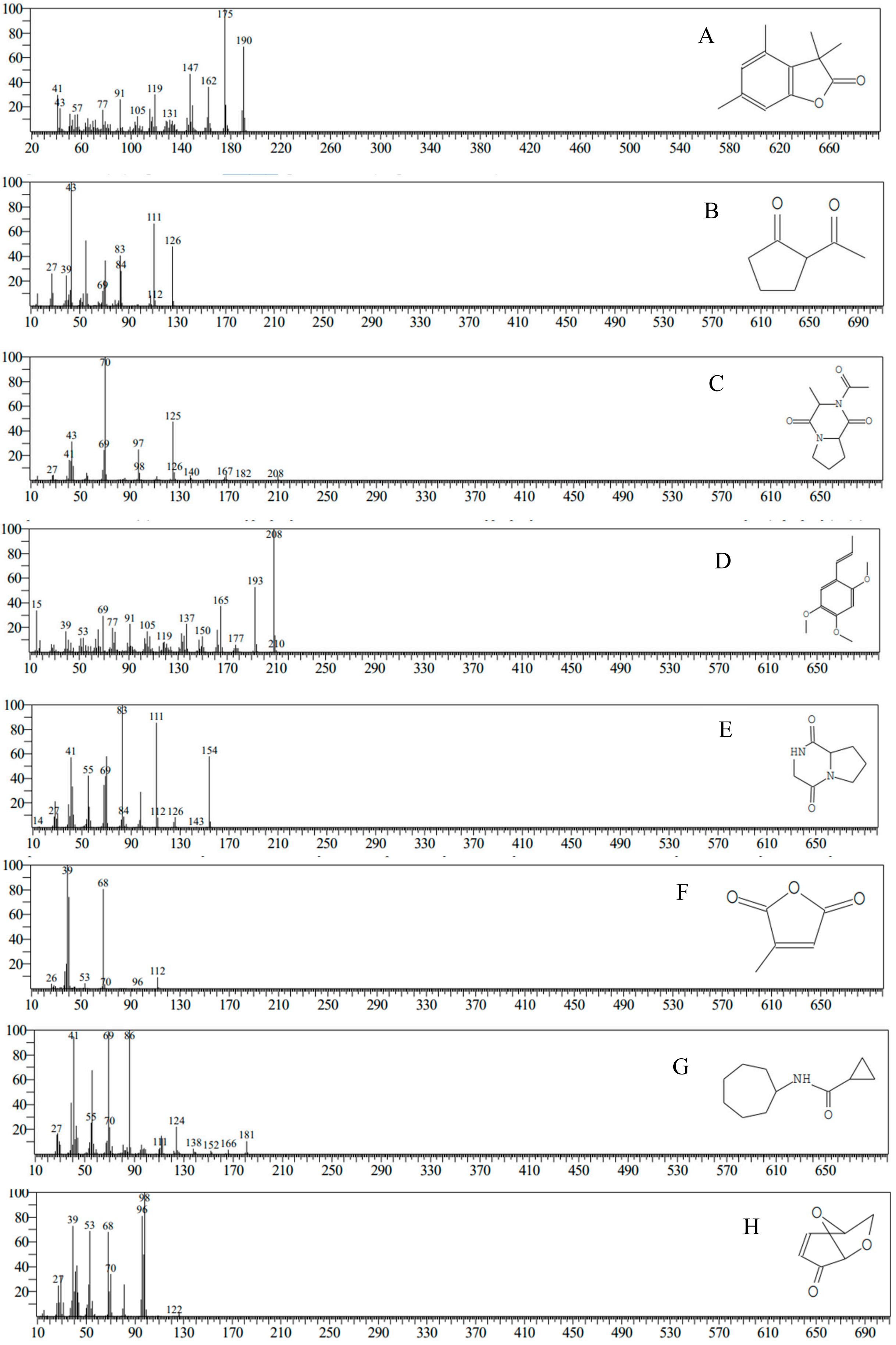

A
41 43 57 77 91 105 119 131 147 162 175 190
B
27 39 43 69 83 84 111 112 126
C
27 41 43 69 70 97 98 125 126 140 167 182 208
D
15 39 53 69 77 91 105 119 137 150 165 177 193 208 210
E
14 27 41 55 69 83 84 111 112 126 143 154
HN
N
F
26 39 53 68 70 96 112
G
27 41 55 69 70 86 111 124 138 152 166 181
NH
H
27 39 53 68 70 96 98 122

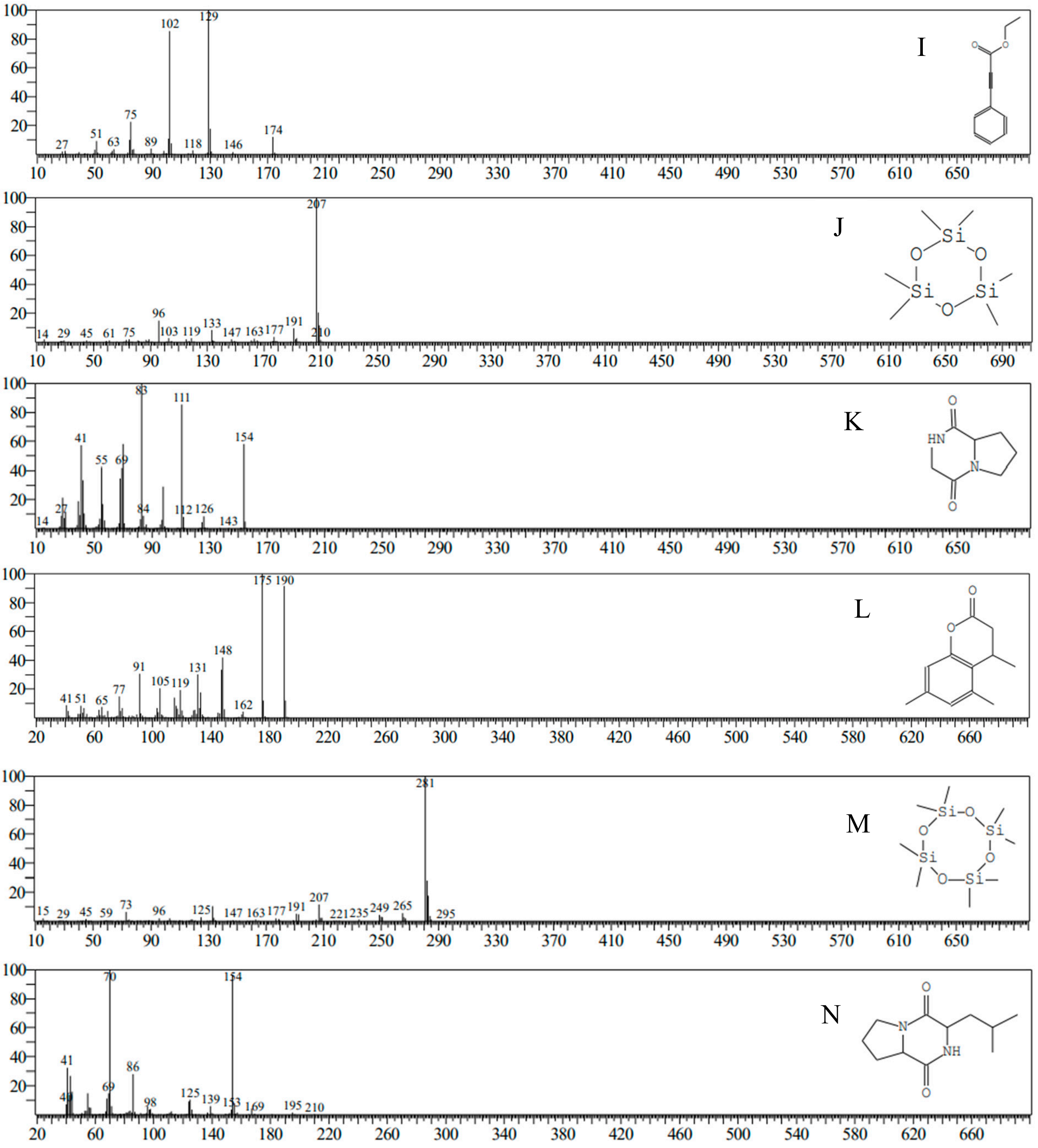

Figure S3: Gas Chromatography-Mass Spectrometry (GC-MS) reports of compounds. GC-MS report of 2,3-2H-Benzofuran-2-one, 3,3,4,6-tetramethyl, peak 190 **(A)**. GC-MS report of 2-Acetylcyclopentanone, peak 126 **(B)**. GC-MS report of 3-Methyl-1,4-diazabicyclo[4.3.0]nonan-2,5-dione, N-acetyl, peak 210 **(C)**. GC-MS report of Asarone, N-acetyl, peak 208 **(D)**. GC-MS report of Pyrrolo[1,2-a]pyrazine-1,4-dione, hexahydro, peak 154 **(E)**. GC-MS report of Citraconic acid, peak 112 **(F)**. GC-MS report of Cyclopropanecarboxamide, N-cycloheptyl, peak 181 **(G)**. GC-MS report of Levoglucosenone, peak 126 **(H)**. GC-MS report of Ethylphenylpropiolate, peak 174 **(I)**. GC-MS report of Hexamethylcyclotrisiloxane, peak 222 **(J)**. GC-MS report of hexahydropyrrolo[1,2-a]pyrazine-1,4-dione, peak 254 **(K)**. GC-MS report of Coumarin, 3,4-dihydro-4,5,7-trimethyl,

peak 190 **(L)**. GC-MS report of Cyclotetrasiloxane, octamethyl-, peak 296 **(M)**. GC-MS report of Pyrrolo[1,2-a]pyrazine-1,4-dione, hexahydro-3-(2-methylpropyl), peak 210 **(N)**.
